# An Unusual Case of Post-Traumatic Headache Complicated by Intracranial Hypotension

**DOI:** 10.3390/brainsci7010003

**Published:** 2016-12-29

**Authors:** Sara Siavoshi, Carrie Dougherty, Jessica Ailani, Kaustubh Yadwadkar, Frank Berkowitz

**Affiliations:** Department of Neurology, Georgetown University Hospital, Washington, DC 20007, USA; Carrie.O.Dougherty@gunet.georgetown.edu (C.D.); Jessica.x.ailani@gunet.georgetown.edu (J.A.); Kyadwadk@gmail.com (K.Y.); FXB10@gunet.georgetown.edu (F.B.)

**Keywords:** post-traumatic headache, intracranial hypotension, syringomyelia, cerebral spinal fluid leak

## Abstract

We present a case of post-traumatic headache complicated by intracranial hypotension resulting in an acquired Chiari malformation and myelopathy with syringomyelia. This constellation of findings suggest a possible series of events that started with a traumatic cerebral spinal fluid (CSF) leak, followed by descent of the cerebellar tonsils and disruption of CSF circulation that caused spinal cord swelling and syrinx. This unusual presentation of post-traumatic headache highlights the varying presentations and the potential sequelae of intracranial hypotension. In addition, the delayed onset of upper motor neuron symptoms along with initially normal head computerized tomography scan (CT) findings, beg the question of whether or not a post-traumatic headache warrants earlier magnetic resonance imaging (MRI).

## 1. Presentation

A 27 year-old man, previously healthy and without history of headache, presented for evaluation of persistent headache seven months after suffering blunt trauma to the back of the head. There was no associated loss of consciousness. He developed a headache the day after injury, which was later accompanied by nausea and vomiting. The headache was described as dull occipital pain with intermittent severe throbbing. Head CT performed one week after the initial injury was unremarkable. At the time of presentation, he described his headache as daily, lasting three to four hours per day with partial pain relief using combination aspirin-acetaminophen-caffeine. His headache was alleviated by lying down and sleeping; and was exacerbated by coughing, lack of sleep and alcohol consumption. Prior to presentation to our clinic, the patient had unsuccessful treatment with amitriptyline, propranolol, sumatriptan, and hydrocodone-acetaminophen.

Past medical and surgical histories were unremarkable. There was no family history of migraine, neurologic disease, or collagen vascular disease. He was a former smoker and reported infrequent alcohol consumption. Caffeine intake included two to three sodas daily.

General and neurologic physical exam were unremarkable. Initial diagnosis was of chronic post-traumatic headache with a chronic migraine phenotype and possible component of medication overuse headache due to frequent use of combination analgesics. He was started on preventive treatment with venlafaxine and instructed to reduce his abortive medication use and caffeine intake.

**Figure 1:**
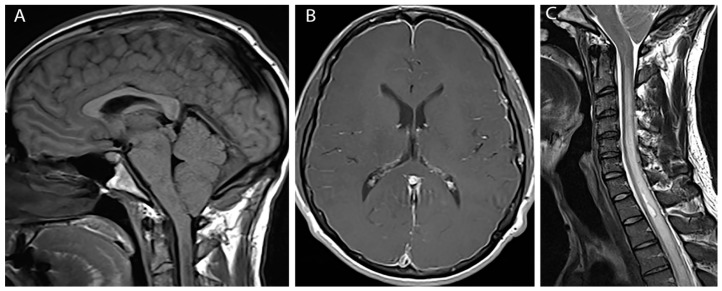
Pre-treatment images (**A**) Sagittal T1 weighted image shows herniation of cerebellar tonsils below foramen magnum; (**B**) Axial post gadolinium image shows diffuse pachymengeal enhancement; (**C**) Sagittal T2 weighted image shows extensive cord edema and syrinx.

At three month follow up the patient reported modest improvement in headache symptoms. However, he had subsequently developed bilateral hand weakness and upper thoracic back pain. Physical exam was notable for bilateral finger flexor weakness, intrinsic hand muscle wasting, tongue fasciculations and ankle clonus. MRI of the brain and cervical cord with and without contrast revealed diffuse pachymeningeal enhancement, sagging brainstem, low lying cerebellar tonsils (10 mm descent) with crowding at the foramen magnum, and venous engorgement of the cervical epidural space (Figure 1A,B). This constellation of findings was suggestive of intracranial hypotension. Additionally, there was diffuse spinal cord signal abnormality involving cervical and upper thoracic spinal cord to the level of T8-T9 with a small syrinx at T1-T2 and an epidural fluid collection in the mid-thoracic spine (Figure 1C).

**Figure 2:**
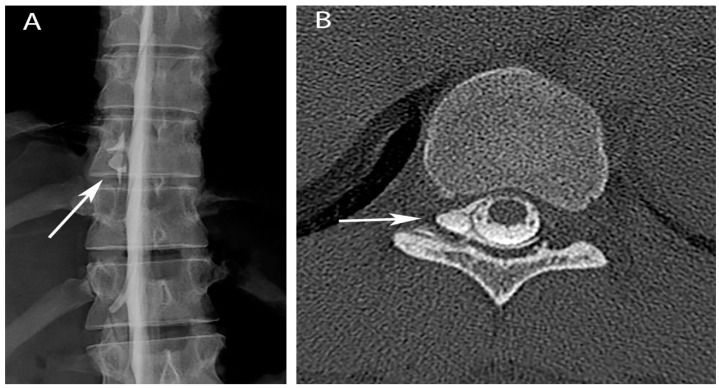
Myelogram (**A**) and post-myelogram CT (**B**) show extravasation of contrast (arrows) at the T10-11 neural foramen (right sided extravasation due to patient positioning).

Upon reviewing the MRI findings with the patient, he confirmed that the headache had always been alleviated by lying supine. A CT guided epidural blood patch was performed at L1/L2, and resulted in only modest improvement in headache pain. A second epidural blood patch was performed at T12/L1, again without significant clinical improvement. Repeat MRI of the brain and spine showed progression of spinal cord signal abnormality from T9 to T10. A CT myelogram showed an epidural fluid collection along the right lateral thecal sac extending from T5 to T12, most prominent at T10 (Figure 2A). CT-guided epidural blood patch directed at T9/T10 resulted in only ephemeral relief. A repeat CT myelogram was performed and a targeted interlaminar epidural blood patch was placed at T9/T10 combined with a right transforaminal blood patch at T10/11. This, the fourth epidural blood patch, also only transiently alleviated the patient’s symptoms. The patient was subsequently evaluated for possible surgical repair of the dural tear. Upon the recommendation of neurosurgery, a myelogram with rapid sequence imaging was performed under biplanar fluoroscopy. This study showed CSF egress from the right T10 nerve root sleeve in the right T10-11 foramen extending into the ventral epidural space (Figure 2B). Repeat combined interlaminar and transforaminal epidural blood patch ultimately resulted in resolution of the patient’s headache. 

**Figure 3:**
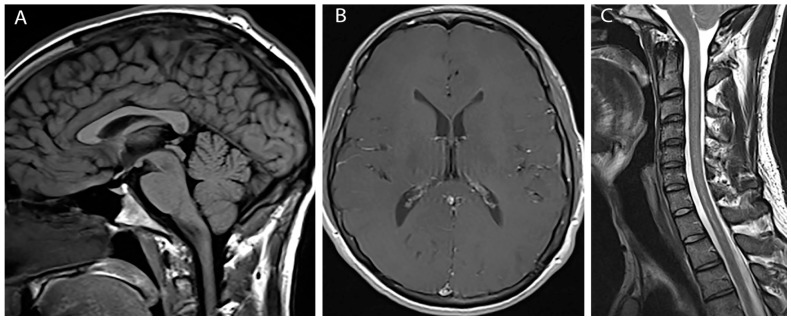
Post-treatment images (**A**) Sagittal T1 weighted image shows resolution of tonsillar herniation; (**B**) Axial post gadolinium image shows resolution of pachymengeal enhancement; (**C**) Sagittal T2 weighted image shows resolution of cord edema and syrinx.

The patient continues to have improvement of bilateral hand strength. His headaches, neck, and back pain all resolved over the following month. Repeat imaging performed approximately three months following the fifth and final therapeutic blood patch showed complete resolution of pachymeningeal enhancement, tonsillar herniation and cord edema with only a tiny residual syrinx (Figure 3).

## 2. Discussion

This case presents several unusual complications of post-traumatic headache. Curiously, the patient developed delayed symptoms that were found to be a result of upper thoracic syrinx and cord edema. 

The positional nature of the headache and exacerbation by coughing were consistent with that of intracranial hypotension [1]. Etiologies of CSF volume depletion include trauma, shunt over drainage, and spontaneous causes, including disorders of connective tissue matrix [2]. The history of blunt trauma to the back of the head in this case would make trauma the most likely cause of CSF leakage. Intracranial hypotension resulting from CSF leakage has been described as a mechanism of acquired Chiari malformation. As spinal fluid is lost, there is loss of brain buoyancy resulting in brain settling and herniation of hindbrain structures through the foramen magnum [3,4].

Although syrinx formation has somewhat of an elusive pathophysiology, several theories have been developed to explain its cause. One of such proposes that in accordance with the Bernoulli theorem, the narrowed flow created by sagging cerebellar tonsils at the foramen magnum, causes an increase in CSF velocity and a resultant low CSF pressure in the narrowed canal. This low CSF pressure creates a suction effect on the spinal cord that distends the cord during each systole, causing extracellular fluid to develop within the distended cord, enlarging the central canal to form a syrinx [5,6]. This can occur in the setting of Chiari malformation and a similar mechanism may occur with trauma [5]. Trauma alone has been well described as a cause for syrinx formation [7,8,9,10,11,12]. In the unique case of our patient described here however, the situation is more complex. It is possible that trauma alone was enough to cause syrinx formation. However, given headache symptoms consistent with that of intracranial hypotension and persistent CSF leakage found on imaging, it is more likely that CSF leakage was the inciting factor. 

We propose that trauma was the cause of CSF leak that led to intracranial hypotension and sagging of cerebellar tonsils, causing distention of the cord and culminating in syrinx formation. An alternative theory that may better explain the delayed onset of weakness is that the patient sustained a second traumatic event that enlarged an initially small dural leak into a larger tear. The larger tear may have resulted in more CSF leakage and caused occlusion of CSF flow at the foramen magnum with the descent of the cerebellar tonsils, ultimately resulting in cord edema and syrinx. A more rudimentary explanation may be that the events occurred semi-independently and not as a causal sequence which we have described. Although the initial head CT did not show evidence of pathology, it is possible the lack of sensitivity CT has for detecting these processes concealed a pre-existing chiari and/or syrinx. In this scenario, a traumatic CSF leaf would have disrupted CSF dynamics and resulted in intracranial hypotension.

Known trauma was most likely to be the precipitating event in this presentation. However, in other cases of spontaneous CSF leak, connective tissue diseases leading to weakness of the dural sac may be considered a potential cause. Studies showing dural ectasia and meningeal diverticula to be common in connective tissue disorders may predispose this population to spontaneous CSF leaks [13,14]. A family history of spontaneous CSF leakage, history of aortic aneurysm, or joint hypermobility on exam may warrant an investigation to look for collagen vascular disease. 

## 3. Conclusions

This case report highlights the importance of correctly identifying headaches associated with a CSF leak. Our young healthy man who presented with a seemingly straightforward case of post-traumatic headache developed delayed synrix formation that resulted in intrinsic hand weakness. CSF leakage is not a benign condition. Potential sequelae including syrinx formation can cause profound debilitation and prove to be quite challenging to treat. Given the risk of the afore-mentioned complications, it is vital a detailed history of the headache characteristics be obtained as well as a thorough neurologic examination performed. Should symptoms or signs consistent with intracranial hypotension be elicited, MR imaging may be warranted.
